# Longitudinal evaluation of dementia care in German nursing homes: the “DemenzMonitor” study protocol

**DOI:** 10.1186/1471-2318-13-123

**Published:** 2013-11-15

**Authors:** Rebecca Palm, Kerstin Köhler, Christian GG Schwab, Sabine Bartholomeyczik, Bernhard Holle

**Affiliations:** 1 German Centre for Neurodegenerative Diseases (DZNE), Stockumer Str. 12, 48453, Witten, Germany; 2 Witten/Herdecke University (UW/H), Faculty of Health, School of Nursing Science, Stockumer Str. 12, 48453, Witten, Germany

## Abstract

**Background:**

In Germany, the number of people with dementia living in nursing homes is rapidly increasing. Providing adequate care for their special needs is a challenge for institutions and their staff members. Because of the growing number of people with dementia, changes to the conceptual orientation of nursing homes have occurred. These changes include specialized living arrangements and psychosocial interventions recommended for people with dementia. Until now, the provision of dementia care and its association to the residents’ behavior and quality of life is not well investigated in Germany. The purpose of this study is to describe the provision of dementia care and to identify resident- as well as facility-related factors associated with residents behavior and quality of life.

**Methods/Design:**

The DemenzMonitor study is designed as a longitudinal study that is repeated annually. Data will be derived from a convenience sample consisting of nursing homes across Germany. For the data collection, three questionnaires have been developed that measure information on the level of the nursing home, the living units, and the residents. Data collection will be performed by staff members from the nursing homes. The data collection procedure will be supervised by a study coordinator who is trained by the research team. Data analysis will be performed on each data level using appropriate techniques for descriptions and comparisons as well as longitudinal regression analysis.

**Discussion:**

The DemenzMonitor is the first study in Germany that assesses how dementia care is provided in nursing homes with respect to living arrangements and recommended interventions. This study links the acquired data with residents’ outcome measurements, making it possible to evaluate different aspects and concepts of care.

## Background

In Germany, up to 70% of care-dependent people with dementia live in nursing homes [1]. Over the last decade, care for people with dementia in nursing homes has changed [2-4]. Modern nursing homes provide a more home-like environment and more privacy for residents [5]. The former traditional medical model is being replaced by a more holistic and person-centered approach which aims at meeting residents' individual needs and enhancing their quality of life [4]. Two core aspects of dementia care concepts in nursing homes are living arrangements and dementia specific interventions.

For Germany, research-based guidelines for the care of people with dementia and challenging behavior in nursing homes are available since 2007 [6]. Several researchers developed the guideline based on a literature review and expert consensus. They recommend the creation of a dementia-friendly environment and architecture, possibilities for further education for nurses, and various forms of living arrangements for residents with dementia. For nursing care, recommendations include psychosocial interventions (validation, multisensory stimulation, reminiscence therapy, physical activities) as well as diagnostics and behavioral assessments. Although the guidelines are not compulsory, they can be considered as a document of high priority for nursing homes. In addition to addressing the residents’ behavior, the overall aim of the guideline is to improve residents' quality of life and well-being.

Today, German nursing homes differ with respect to living arrangements. Two forms exist: integrative and segregative. The principle of integration allows residents with and without dementia to live together; segregation includes several forms of specialized living arrangements exclusively for residents with dementia (e.g., Dementia Special Care Units [DSCU], small living units with home-like environment). Regarding dementia specific interventions, a great effort has been invested in developing, testing and implementing such interventions in Germany [7].

In the last decade, national as well as international studies evaluated the implementation and the effect of dementia specific living arrangements and interventions on resident’s behavior and quality of life.

### Evaluation of living arrangements

The effect of DSCU residence on residents quality of life was investigated in a large survey study from the United States involving 390 nursing homes and 13,983 residents [8]. The results of this study indicated a positive relationship between quality of life and residence in a DSCU. Moreover, this study showed that facility characteristics play an important role in resident perception of quality of life [8]. A study from Spain [9] showed contradictory results: the staff rated residents’ quality of life on DSCU’s lower than of residents living in a regular unit. For Germany, no study exists that applied a quality of life measurement. The only study that evaluates DSCU’s in Germany investigated indicators for quality of life such as social contacts and activities [10]. They found benefits to living in a DSCU compared to traditional units. Concerning challenging behavior, the study did not show a beneficial effect of DSCU placement, a result that is confirmed by the latest Cochrane Review [11].

Regarding small-scale living arrangements a German study did not show a clear effect on residents quality of life or on behavioral problems [12]. Studies from the Netherlands and Belgium reported the same findings [13,14].

Conclusions drawn from these studies suggest that the implementation of best practices may be more important than providing a specialized environment [11] and that future research should focus more on the quality and content of care than on the scale or specialization for the evaluation of quality of life [14].

### Evaluation of quality of care

Concerning the implementation of guidelines for psychosocial interventions in dementia care, a comparison of seven European guidelines, including the German version, showed weaknesses in their applicability [15]. The latest review on nonpharmacological interventions concluded that the feasibility of the investigated interventions is limited because of resource requirements [16]. Recently, a set of 12 quality indicators for psychosocial care in dementia in nursing homes was developed as part of the European Collaboration on Dementia project (EuroCoDe), which was initiated by Alzheimer Europe [17]. The results from the first application draw a different picture than what may was suspected. They indicate that the majority of residents receive psychosocial interventions tailored to the person’s needs and abilities (this was found for 50% of residents with dementia in Dutch nursing homes and 97% in Spanish nursing homes), that the residents documented care plan included different forms of activity (100% of the Dutch sample and 21% of the Spanish sample) and that – at least in the Netherlands – 75% of the residents with dementia and behavioral problems are treated with a psychosocial intervention first [17]. But these results have to be interpreted cautiously taking the limitations into account: the discriminatory capacity is not yet assessed and the assessment of the indicators relies on documentation only [17].

There are few studies reporting on the quality of care in specialized units compared to traditional units. Studies have investigated differences in care processes in dementia special care units compared to traditional units [10,18,19], but these studies mainly focused on clinical process measures (e.g., feeding tube use, physical restraint use, psychotropic medication use, and incontinence care) whereas the use of dementia specific interventions remains unclear. Only Weyerer et al. [10] report more participation in physical activities, activities in and outside the nursing home, memory training and biography-oriented groups/individual sessions for DSCU’s compared to traditional units.

In summary, a large amount of research has been conducted to evaluate current approaches to dementia care in nursing homes. But still a knowledge gap exists regarding details of how dementia care is provided in practice, which type of interventions are in use, how these interventions are related to existing institutional resources and resident characteristics and how facility characteristics, such as size, ownership type, staffing levels and the provision of dementia specific interventions influence resident’s behavior and quality of life [20-25].

Explorative knowledge about the provision of institutional care for people with dementia and the factors that are associated with quality of life and behavior is necessary for future intervention research and the further development of quality indicators to base policy decisions on sound scientific evidence.

#### Aims and research questions of the study

We have designed a longitudinal study called the “DemenzMonitor”. The overarching aim of the study is to identify resident- and facility-related factors and covariates that are associated with

a) the residents behavior and

b) the residents quality of life.

To reach this aim, the following research questions will be answered:

1. How is dementia care provided in German nursing homes? Which living arrangements are in use? Which recommended interventions are in use? Who gets which interventions?

We assume that there will be differences with respect to the care provided between segregative and integrative living units, as well as small- and large-scale units. Therefore, we will investigate these as fixed groups. Based on certain other key variables (ownership, costs, special reimbursements, staff) we aim to build further possible groups with similar characteristics.

2. Are there any differences between the groups regarding the resident’s demographic data, cognition, care dependency? Are there any differences between the groups regarding the interventions provided?

For the whole population as well as for the groups we seek to answer this question:

3. Which of the investigated variables are associated with the resident’s outcomes behavior and quality of life when controlling for resident-related covariates such as age, sex, length of stay? Are there differences between the results of the whole population and the groups?

Since the study provides longitudinal data, we will also be able to answer the questions:

4. Are the resident’s outcomes behavior and quality of life stable over time or are they changing? If they change, are there differences in associated factors and covariates between the two points of time so that time needs to be considered as an influencing factor? Which factors are associated with the change of the resident’s outcomes?

Based on these results, hypothesis on influencing factors of resident’s behavior and quality of life will be generated.

## Methods

### Design

The DemenzMonitor is intended to be an ongoing observational descriptive longitudinal study to be repeated every year.

### Study population and recruitment

Nursing homes across Germany are invited to participate in the study. In 2011, more than 12,000 nursing homes existed in Germany [26]. Because it is not feasible to contact every institution, the study will be published in high-circulation professional journals, newsletters, and the websites of nursing and geriatric information services. It will also be presented at national nursing conferences. It is assumed that the motivation of nursing homes to participate will be strongly driven by the benefits they will receive from the study. Therefore, the participating institutions will receive an individual report with living-unit- and resident-related results. The template for this report was developed together with institutions from the pilot study to ensure practicability (see “Dissemination of study results to the participating institutions”).

Accurate data collection requires a considerable amount of time and motivation by participating institutions and their employees; therefore, participation is voluntary. If an institution chooses to participate, it is that institution’s responsibility to determine how many living units will be involved. If informed consent is given, we propose a whole-population survey of the participating living units. The goal is to involve the institutions for as long as possible to gather longitudinal data. However, because participation is voluntary, the institutions will decide how long they will participate and how many data collections will be performed. It is assumed that a number of institutions will decline to participate repeatedly. Therefore, new institutions will be recruited and involved each year.

After each data collection cycle, recruitment rates for the proportion of participating institutions in every federal state will be calculated to get an idea of representativeness.

### Conceptual framework

The study is based on a self-developed conceptual framework that guided its design, development, and the selection of the range of potential determinants of residents’ outcomes. The framework is based on the concept of multi-level social epidemiology frameworks [27]. These frameworks arose from a critical discussion about the limitations of epidemiology’s dominant causal models and views. In this paradigm, causality is assumed to be linear with proximate, individual risk factors, whereas social epidemiology frameworks account for the joint and dynamic influence of social, environmental, and biological factors that affect health [28]. “Eco-epidemiology”, a framework proposed by Mervyn Susser in 1996 [29], encompasses multiple interactive systems at different levels. Eco-epidemiology is grounded in the principle of ecologism, which seeks to understand phenomena in relation to the boundaries of context rather than seeking universal explanations that may be context-free [30].

**Figure 1:**
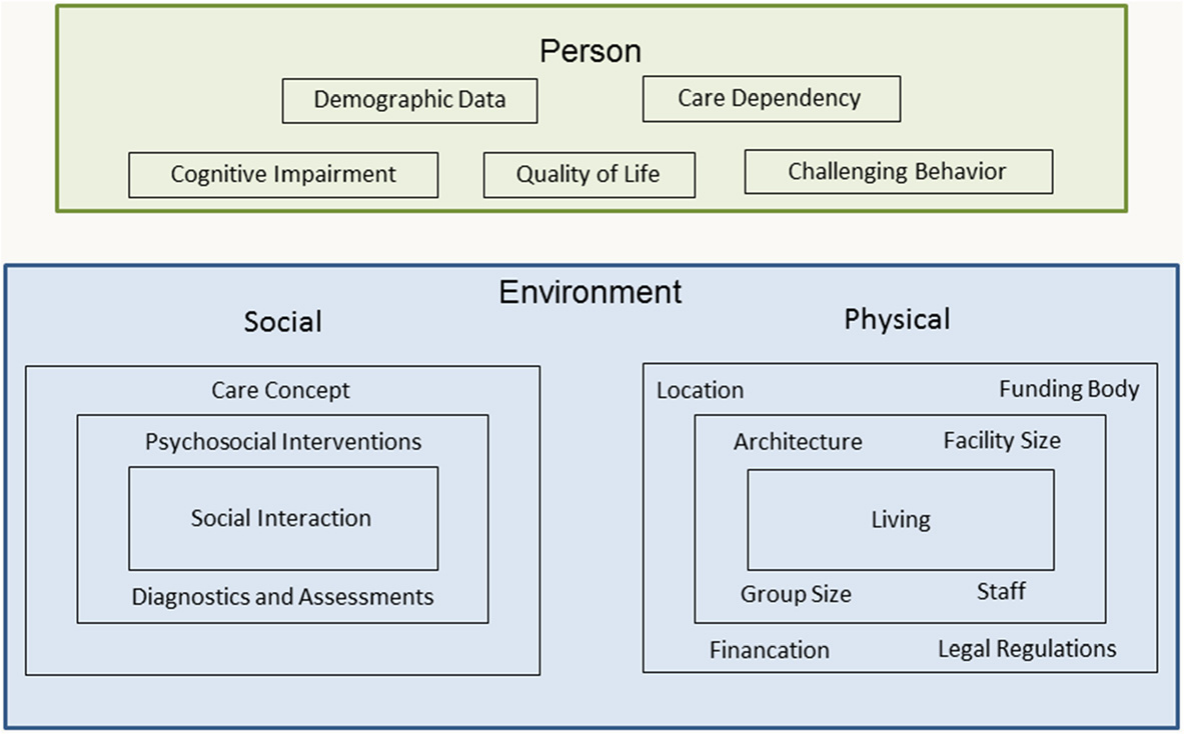
Conceptual framework of the study.

The framework developed for the study refers to the work of Lawton and Nahemow [31,32], which focuses on behavior and quality of life. The central thesis of this work is that competencies of the individual, the environment, and the interaction of the individual with the environment influence human behavior and quality of life. According to ecological frameworks, the DemenzMonitor framework relies on two dimensions to clarify the complexity of social realities: environment- and person-focused dimensions. The environment-focused dimension comprises physical and social environments. The person-focused dimension includes demographics, function, and dementia-specific characteristics such as cognition, behavior, and quality of life. Figure 1 shows the conceptual framework.

### Instrument development

To assess the different dimensions, it was necessary to develop a new instrument that covers three levels: the level of the institution, the living units, and the residents.

Based on the conceptual framework, we conducted a literature search to identify existing instruments or measurements that are suitable to assess the different aspects of the dimensions. This search identified instruments for person-related aspects (care dependency, cognitive impairment, challenging behavior, quality of life). As we did not find any suitable instruments relating to the aspects of the social and physical environment dimension, new items had to be developed. The development of these items was conceptually based on the German guidelines for the care of people with dementia and challenging behavior in nursing homes [6]. Additionally, we conducted a systematic literature review on the development, implementation and use of this interventions in the practice [7]. Focus groups and quantitative expert ratings were also conducted to achieve content and face validity [33,34]. A multi-method pretest with intended users revealed problems with comprehensibility and practicability [35,36].

Each step of the instrument development and testing followed a revision of the instrument. The developed instrument was applied in a pilot study in May 2012.

### Questionnaires

The new instrument contains three questionnaires divided into several sections. An overview of the sections for every questionnaire provides Table 1.

**Table 1:** Overview of the questionnaires

	**Items on each level**		
**Section**	**Institution**	**Living unit**	**Resident**
General characteristics	37	4	17
Staff characteristics	27	42	/
Characteristics of living environment	6	19	3
Living and care concept	31	14	/
Provision of dementia care	/	38	80
Care dependency (PSMS)	/	/	6
Challenging behaviour (NPI-Q)	/	/	24
Dementia (Diagnosis)	/	/	1
Dementia (FAST)	/	/	16
Dementia (DSS)	/	/	7
Quality of Life (Qualidem)	/	/	40
Total	101	117	194

At both the nursing home and living unit levels, the questionnaires focus on environmental aspects (e.g., general and staff characteristics, characteristics of the living environment, living and care concepts). The provision of care is operationalized at the level of the living unit and the residents. Additionally, several assessment instruments are included in the residents’ questionnaire, as explained below.

Tables 2, 3 and 4 provide an overview of the questionnaires. The questionnaires can be obtained on request.

**Table 2:** Measurements on nursing home level

**Nursing Homes [NH] - assessed by nursing home mangers**			
**Conceptual domain**	**Operational definition**	**Number of items**	**Empirical measure**
General characteristics	Funding body	1	3-response option
	Locality measured in inhabitants	1	4-response option
	Location (federal state)	1	16-response option
	Number of beds in long term care / respite care / day care / night care / residential care	5	Free-text
	Number of residents per care level	5	Free-text
	Number of residents per care level with substantial additional care needs according to Long Term Care Insurance	5	Free-text
	Costs for care per care level / accommodation & expenditure (single/double room)	9	Free-text
	Existence of a segregative living unit (SLU) with additional costs	1	Yes/No
	Costs for the SLU for care per care level / accommodation & expenditure (single/double room)	9	Free-text
Staff characteristics	Staff divided in subgroups (administrative staff / registered nurses / nursing assistants / nursing students / additional care staff / facilitating services)	13	Fulltime equivalent (planned)
	Number of volunteers	1	Free-text
	Number of engaged clinical nurse specialists for Psychogeriatrics / Psychiatry / Palliative care / Dementia Care Mapping (Basic User) / multisensory stimulation / validation therapy / other dementia-related trainings	7	Free-text
	Training of at least half of all nurses employed in case conferences / behavioral cognition assessments / validation therapy / reminiscence therapy / multisensory stimulation / physical activity	6	Yes/No
Characteristics of living environment	Year of nursing home (NH) foundation	1	Free-text
	Time span since last modernization	1	6-response option
	Number of living units	1	Free-text
	Number of single / double / multi-shared rooms	3	Free-text
Living and care concept	Solely specialized for dementia	1	Yes/No
	Type of living arrangement (integrative/segregative)	1	2-response option
	Number of segregative living units (SLU)	1	Free-text
	SLU for residents with very severe dementia	1	Yes/No
	Care is based on a written dementia-specific concept	1	Yes/No
	* Concept basis (Person centered care [Tom Kitwood] / Psycho-Biography [Erwin Böhm] / Validation concept [Naomi Feil, Nicole Richard, Cora van der Kooij] / Milieu therapy / Normalization principles / None of these approaches)	7	Yes/ No
	* Characteristics of institutional dementia-specific concept (nurses/assistants are always working on the same LU / engagement of additional staff for daily activities / small scale living [max. 15 residents] / standard for admission procedures / structuring of daily activities / flexible mealtimes / night times / personal hygiene / active involvement of relatives)	12	Choose a maximum of 3
	* Nursing interventions as part of the dementia-specific concept (case conferences / behavioral and cognition assessments / validation therapy / reminiscence therapy / multisensory stimulation / physical activity / others)	7	Yes/No

* conditional answer depending on previous answer.

**Table 3:** Measurements on living unit level

**Unit level (Living Units [LU] – assessed by head nurses)**			
**Conceptual domain**	**Operational definition**	**Number of items**	**Empirical measure**
General characteristics	Number of beds in long term care / respite care	2	Free-text
	Number of residents at day of data collection in long term care / respite care	2	Free-text
Staff characteristics	Staff divided in subgroups working on the ward at certain times (4) (registered nurses (RN) / nursing assistants (NA) / nursing students / additional care staff / facilitating services)	36	Free-text
	RN and NA are constantly allocated to the unit	1	Yes/No
	Service workers are constantly allocated to the unit	1	Yes/No
	Continuous presence of a RN during day shift	1	Yes/No
	Qualification of the head nurse of the unit (Psychogeriatrics / Psychiatry / Palliative care)	3	3-response option
Characteristics of living environment	Number of single / double / multi-shared rooms	3	Free-text
	Dementia specific architecture	1	Yes/No
	Time span since last modernization	1	6-response option
	Permission of residents to bring own furniture	1	Yes/No
	Furnishing of public rooms (more functional / functional and individual / more individual)	1	3-response option
	Permission to bring a pet	1	Yes/No
	Possibility to have contact with animals	1	Yes/No
	Accessible (safe-guarded) outdoor area	1	Yes/No
	Preparation of meals in the living-unit	1	Yes/No
	* Preparation of breakfast / lunch / coffee & tea / dinner / snacks	5	Yes/No
	Meal serving system (tablet / served by staff / buffet / home-like)	3	Yes/No
Living and Care Concept	Living arrangement (integrative/segregative)	1	2-response option
	Structural segregation	1	Yes/No
	Exit control	1	Yes/No
	Special refinancation of segregative living unit (SLU)	1	Yes/No
	* Scope of refinanciation (staff / concept / structure / others)	4	Yes/No
	* Criteria for admission to SLU (diagnosis of dementia / care level / dementia severity / challenging behaviour / mobility / others)	6	Yes/No
Provision of dementiacare	Performance of case conferences (CC)	1	Yes/No
	* Characteristics of CC: CC are following a structured procedure / CC are conducted multidisciplinary / an external moderator is involved if needed / invitation of residents / relatives / results are recorded / results are evaluated / during CC’s staff has no other duties	7	Yes/No
	* Occupational groups invited to CC (residents / relatives / legal guardian / head nurse / nurses / additional care staff / physicians / therapists / others)	9	Yes/No
	* Time schedule of CC	1	3-response option
	* Location of case conference (conference room / nurses office / staff recreation room)	1	3-response option
	* Frequency of disruptions of CC due to work demands on the living unit	1	4-response option
	Snoezelen equipment on the living unit	1	Yes/No
	Performance of physical activities (PA): duration	8	Free-text
	Performance of PA: frequency	8	3-response option
	PA for residents with mobility restrictions	1	Yes/No

* Conditional answer depending on previous answer.

**Table 4:** Measurements on the resident level

**Resident level (assessed by nurses)**			
**Conceptual domain**	**Operational definition**	**Number of items**	**Empirical measure**
General Characteristics	Gender	1	Female/Male
	Date of birth	1	Date
	Place of residence before NH admission	1	5-response option
	Date of entry into nursing home and write NH	1	Date
	Legal guardian	1	4-response option
	Court order for admission	1	Yes/No
	Court order for physical restraints	1	Yes/No
	Care level according to Long Term Care Insurance (LTCI)	1	5-response option
	Substantial additional care needs according to LTCI § 87b	1	3-response option
	Visitors	1	Yes/No
	* Frequency of visits (spouse / other relatives / friends-neighbors / legal guardian / volunteers / other residents / others)	7	4 point-Likert-Scale
Characteristics of Living Environment	Residents room (single / double / multi-shared rooms)	1	3-response option
	Individual furniture in residents room	1	Yes/No
	Resident brought a pet to the NH	1	Yes/No
Provision of Dementia Care	Case conference (CC) after admission	1	Yes/No
	* Date of last CC	1	Date
	* Participants of last CC (resident / relatives / legal guardian / head nurse / ward nurses / other care staff / physicians / therapeutic staff / facilitating service / external moderators / others)	11	Yes/No
	* Reason for conducting last CC (due to an acute occasion / due to the routines)	1	2-response option
	* Content of last CC (nutrition / continence problems / risk of falls-actual falls / chronic wound / physical restraints / acute health problems / pain / cognition based problems / challenging behavior / psychosocial situation / quality of life - well-being / needs of the resident and relatives / admission to NH / hospital stay / others)	15	Yes/No
	Assessment of pain	1	Yes/No
	* Instrument used for pain assessment	1	11-response option
	* Date of pain assessment	1	Date
	Assessment of behavior	1	Yes/No
	* Instrument used for behavioral assessment	1	6-response option
	* Date of behavioral assessment	1	Date
	Assessment of dementia severity	1	Yes/No
	* Instrument used for dementia severity assessment	1	10-response option
	MMSE-Score (if available)	1	Free-text
	* Date of MMSE	1	Date
	Assessment of quality of life	1	Yes/No
	* Instrument used for quality of life assessment	1	6-response option
	* Date of quality of life assessment	1	Date
	Assessment of depression	1	Yes/No
	* Instrument used for depression assessment	1	3-response option
	* Date of depression assessment	1	Date
	Participation in Dementia Care Mapping	1	Yes/No/Unknown
	*Date of last Dementia Care Mapping	1	Date
	Assessment of biography	1	Yes/No
	* Amendment of biography assessment after initial assessment	1	Yes/No/Unknown
	Provision of multisensory stimulation (aroma therapy / music therapy / massage / listening to music / Basale Stimulation^©^ / Snoezelen / cuddling pets / using touch materials / others / none)	10	Yes/No
	Provision of validation therapy	1	Yes/No
	* Kind of validation therapy (use in daily conversation / in personal communications / in group therapy / as a crisis intervention)	4	Yes/No
	Frequency of being in the open air during the last week	1	5 point-Likert-Scale
	Participation on physical activities (PA) (gymnastics / dance / games / walk outside / physiotherapy / others / none)	1	Yes/No
	* Kind of PA (gymnastics / dance / games / walk outside / physiotherapy / others)	10	Yes/No
	Incidence of acute psychiatric crisis in the last 6 months	1	Yes/No/Unknown
	* Frequency of acute psychiatric crisis in the last 6 months	1	4 point-Likert-Scale
	Continuous attendance by a General Practitioner	1	Yes/No
	Continuous attendance by a neurologist/psychiatrist	1	Yes/No
Care Dependency	Physical Self Maintenance Scale (PSMS) [55]	6	5 point-Likert- Scale
Behavior	Neuropsychiatric Inventory (NPI-Q) [48]	12	3 point-Likert-Scale
Dementia	Medical diagnosis of dementia	1	Yes/No/Unknown
	Functional Assessment Staging (FAST) [51]	16	7 stages
	Dementia Screening Scale (DSS) [53]	7	3 point-Likert-Scale
Quality of Life	Qualidem [41]	40	4 point-Likert-Scale

* Conditional answer depending on previous answer.

### Assessments

The residents’ questionnaire includes five assessments described in detail below. All assessments used are proxy-rating instruments and administered by the professional caregiver who is most familiar with the respective residents.

#### Quality of life

Quality of life is a complex and multidimensional concept that is influenced by both individual and environmental factors [37,38]. Moreover, the definition of quality of life involves a subjective component. For this reason, self-reports are considered the gold standard [39,40]. However, communication, memory, and cognitive impairments hamper the evaluation of self-reported quality of life in people with dementia, and the reliability and validity of self-reported quality of life is questioned in the literature [41]. Therefore, specific proxy-rating instruments for people with dementia have been developed. For this study, the quality of life assessment will be conducted using the Qualidem questionnaire, which has been specifically designed and validated [42] for institutionalized residents with dementia over the age of 65 years who suffer from mild to severe dementia. It is available in German and shows satisfactory psychometric properties in the German translation [43,44]. Qualidem assesses nine domains of quality of life, including 37 indicative and contraindicative items with four possible responses (i.e., never, rarely, sometimes, and frequently). Responses to these items determine the subscales: care relationship, positive affect, negative affect, restless or tense behavior, positive self-image, social relations, social isolation, feeling at home, and something to do. In the case of severe dementia (Global Deterioration Scale 7), six subscales can be applied using 18 of the 37 items [45]. To ensure reliability, Qualidem should be administered by two professional caregivers [42]. For this study, the institutions were informed of and requested to follow this recommendation.

#### Challenging behavior

Challenging behavior is also a complex and multidimensional construct [46]. In general, behaviors ranging from aggressive to apathetic are distinguished [6]. Several instruments exist to assess challenging behavior in residents with dementia. The Neuropsychiatric Inventory (NPI-NH) is a widely used instrument to measure neuropsychiatric behavior in dementia research [47]. It comprises 12 domains: delusion, hallucination, depression, anxiety, euphoria, aggression, apathy, disinhibition, irritability, aberrant motor behavior, sleep problems, and eating disorders. For this study, the simplified clinical form of the NPI, the NPI-Q [48], is used. It reports two scores for each domain: the presence of behavior and the severity of behavior on a 0–3 scale (0 = none, 1 = mild, 2 = moderate, 3 = severe). The calculations use either the severity score for each domain or the total score, which ranges from 0 to 36. The clinical form of the NPI was chosen for feasibility reasons.

#### Dementia diagnosis

Data on diagnosis of dementia are obtained from nursing home records.

#### Cognitive impairment

There are several ways to assess cognitive impairment in study participants [49]. A common tool for assessing cognitive function is the Mini Mental Status Examination (MMSE) [50]. For practical reasons, a MMSE cannot be performed for this study. However, in several nursing homes, a MMSE is performed regularly as a standard procedure. Therefore, we assess whether a MMSE value is available for a resident and, if so, when the MMSE was conducted. To gather more information about the cognitive status of residents, two other assessment instruments are part of the DemenzMonitor questionnaire. As a staging scale, the Functional Assessment Staging (FAST) [51] will be applied. Additionally, a new dementia screening instrument is included, the Dementia Screening Scale (DSS) [52,53].

The FAST allows the evaluation of changes in functional performance throughout the entire course of Alzheimer’s disease. It assesses functional capabilities including activities of daily living (ADL) as well as instrumental activities of daily living (IADL). The FAST scale includes seven major functional levels (1–7) operationalized by 16 items that are concordant with the corresponding global level of cognition and functional capacity of the Global Deterioration Scale [54]. The results of psychometric testing indicate that the FAST is a valid and reliable instrument for evaluating functional deterioration in people with Alzheimer’s disease.

The DSS was developed in German, shows satisfactory psychometric properties, and can differentiate among residents with severe dementia [53]. It was chosen because it is a simple and economic screening instrument that can be applied by nurses and is feasible for screening a large number of residents. Furthermore, it allows comparisons with results from national studies. The instrument comprises a series of seven items and includes two domains of cognitive functioning: memory and orientation. Items are rated on a 0–2 scale (0 = always, 1 = sometimes, 2 = never). The total score ranges from 0 to 14; a higher score indicates stronger impairment.

#### Functional status

To indicate the functional status of the residents, the Physical Self-Maintenance Scale (PSMS) [55] was chosen. The PSMS assesses self-maintaining and instrumental activities of daily living, such as (in)continence, requiring assistance with feeding, getting dressed, personal hygiene, mobility, and bathing. The items are rated on a 1–5 scale, with more points indicating greater dependency. The PSMS is a valid and reliable measure [55] and is recommended on the basis of an expert consensus [56].

### Ethical considerations

The health care staff will collect data from residents. Therefore, written informed consent must be secured. The residents or their registered legal representatives must be informed of the purpose of the study and the conditions of participation. In terms of data security the residents’ identity will be kept confidential by using a pseudonym (code) for the questionnaires. The code list with the names of the residents will be stored in the nursing homes. The researchers are not going to have access to the list.

The ethics committee of the German society of Nursing Science approved the study.

### Procedure for data collection

Every participating institution must designate a study coordinator to be the contact person for the researchers. The study coordinator is responsible for the entire data collection process, which includes informing and educating all persons involved in the study (nurses and residents), selecting the persons who will collect the data, briefing and supporting these persons, codifying the living units and residents, and disseminating the results within the institution. The research team will prepare the study coordinators for this task. The coordinators will be trained during a one-day lecture on the data collection procedures that focuses particularly on the questionnaires. Furthermore, the research team will provide the coordinators with all necessary material, including brochures and letters.

If a study coordinator leaves the institution but the institution continues to participate, a new study coordinator will be designated and trained.

The data collection will be conducted within a fixed time period of one month. Individual institutions will decide when to collect the data during this period.

### Data entry, coding, cleaning, and storage

There are two ways of entering the data: online or paper-pencil. Secured online data collection will be conducted using the web tool LimeSurvey and the research center’s own server. Both data sets will be merged into a self-developed database and processed offline.

The study coordinators will code the residents’ questionnaires. To generate an individual code they will use constant items related to the resident (i.e., number of the nursing home and living unit, sex, date of birth, and first and last letter of the family name). Correct coding is important when collecting longitudinal data; therefore, we developed a technique that enables us to correct the code if necessary. With the help of stable items (e.g., date of entry), a matching method will indicate highly similar cases. This process is able to detect typing errors or mistakes in the coding such that manual error handling is possible. A detailed description of this technique is described elsewhere [57].

Data will be checked for missing items, plausibility, and the observance of determined time periods required to correctly complete the assessment instruments. Missing items will be imputed where appropriate. Inconsistent items will be removed.

For longitudinal analysis data sets will be checked for drop outs. Missing data due to drop-outs of the nursing home or death of the resident will not further be considered in the longitudinal analysis.

Data will be saved in a demilitarized zone of the research center for ten years. The paper-and-pencil questionnaires will be scanned and saved in the same manner.

### Data analysis

Since the nature of the study is explorative, predominantly data mining techniques such as visualization of data, classifications of data, association and regression analysis will be applied. To answer the first research question, frequency analysis will be computed for the data from the whole study population. An appropriate classification technique (e.g. cluster analysis) will be conducted to identify possible groups. To answer the second research question descriptive statistics will be applied. Performing association and regression analysis will answer the third and fourth research question. To account for the nested structure of data due to different levels of data and time points, mixed effects generalized linear models will be used.

### Dissemination of study results to the participating institutions

The research team will fed back the residents results for practical use. Therefore, a self-developed Access^©^ database will automatically generate a report for every participating institution. This report will contain the results of the assessments for each resident as well as aggregated resident results for every living unit. For each resident, the report will display results of the DSS, NPI-Q, PSMS and Qualidem graphically. Table 5 displays the reported indicators on the living unit level. The report will also contain the average results for the whole sample as a benchmark for every indicator. The indicators will be for practical use only and will not be validated for scientific use at the time of their dissemination.

**Table 5:** Indicators included in feedback reports

**Numerator**	**Denominator**^**a**^
**Assessment**	
1. Number of residents who are assessed for pain during the last 4 weeks / 3 months / > 3 months	Total number of residents
2. Number of residents who are assessed for behavior during the last 4 weeks / 3 months / > 3 months	Total number of residents
3. Number of residents who are assessed for dementia severity	Number of residents with a medical diagnosis of dementia
4. Number of residents who are assessed for depression during the last 4 weeks / 3 months / > 3 months	Total number of residents
5. Number of residents who are assessed for quality of life during the last 4 weeks / 3 months / > 3 months	Total number of residents
**Recommended interventions**	
6. Number of residents for whom a case conference was conducted since he/she moved in	Total number of residents
7. Number of residents who received multisensory stimulation interventions	
8. Number of residents who received validation therapy	
9. Number of residents who were daily in the open air during the last week	
10. Number of residents who were not at all in the open air during the last week	
11. Number of residents who participated in a physical activity	
12. Number of residents who received an intervention for managing an acute psychiatric crisis during the last six months	
**Behavioral problems**	
13. Number of residents with delusions	Total number of residents
14. Number of residents with hallucinations	
15. Number of residents with depression	
16. Number of residents with anxiety	
17. Number of residents with euphoria	
18. Number of residents with aggression/agitation	
19. Number of residents with apathy	
20. Number of residents with disinhibition	
21. Number of residents with irritability	
22. Number of residents with aberrant motor behavior	
23. Number of residents with problematic nightly behavior	
24. Number of residents with problematic eating behavior	
**Dementia**	
25. Number of residents with a medical diagnosis of dementia	Total number of residents
26. Number of residents with dementia according to DSS	
27. Number of residents with dementia according to FAST	

^a^ The total number refers to the number of residents with complete data for this item.

## Discussion

The DemenzMonitor is the first study in Germany to assess how dementia care is provided in nursing homes with respect to structural conditions and psychosocial interventions and to link these data with residents’ outcome measurements.

The study is intended to be repeated every year to facilitate long term observations. Yearly repetition will allow structural changes in the nursing home care sector to be monitored, and longitudinal data will allow the investigation of relations without experimental manipulation.

The newly developed questionnaires allow the characterization of participating institutions in multiple ways. Given the broad variety of facilities, it will be possible to evaluate different aspects of care and the influence of resident and facility characteristics. Because the questionnaires are partly based on the German guideline and only parts are used internationally, conclusions drawn from the study will apply primarily to the German long term care system. However, the results of the study will contribute to the exploration of complex residents’ outcomes, such as quality of life and challenging behavior by adding to the body of literature on this topic [58-63]. The longitudinal approach of the study will allow for the observation of changes and factors associated with changes in residents’ outcome measures and the exploration of variations in resident outcomes. Understanding the nature of change is important for the development of interventions and the identification of residents who most likely will benefit from them [64].

The knowledge derived from this study is also important for the further development of quality tools, including national guidelines and instruments to enhance the quality of care, such as quality indicators. Moreover, this study will allow for a deeper understanding of which interventions should be recommended for whom and when. Regarding the methodological development of quality indicators, this study will contribute information on what type of self-reported data should be considered as a data source for quality indicators. Further testing of the developed questionnaire would be required to determine the validity and reliability.

### Limitations

This study design has certain limitations that restrict the generalizability of the results. Because data are derived from a convenience sample, the results are limited in their representativeness. Once the study is established and experience is gained concerning the data collection procedures, changes in the sampling strategy will be considered. Given this recruitment approach, the results from the study must be interpreted cautiously. Best performing institutions may be more willing to participate, which could cause a potential selection bias. Moreover, data concerning the provision of care are collected using staff self-reports, resulting in a potential bias due to social desirability. Concerning the residents’ assessments, proxy-ratings also have methodological constraints. For quality of life, proxy-ratings systematically score lower than self-ratings [65,66] and correlate with caregiver burden [67] and staff attitudes [59,68].

## Competing interests

The authors declare that they have no competing interests.

## Authors’ contributions

RP drafted the manuscript. KK, CS, SB, and BH helped draft this manuscript. All authors contributed to designing the study. All authors read and approved the final manuscript.

## Pre-publication history

The pre-publication history for this paper can be accessed here:

http://www.biomedcentral.com/1471-2318/13/123/prepub
